# Characteristics of the gut microbiota in professional martial arts athletes: A comparison between different competition levels

**DOI:** 10.1371/journal.pone.0226240

**Published:** 2019-12-27

**Authors:** Ru Liang, Shu Zhang, Xiangji Peng, Wanna Yang, Yanwei Xu, Ping Wu, Junhui Chen, Yongjiang Cai, Jiyuan Zhou

**Affiliations:** 1 Institute of Management, Beijing Sport University, Beijing, China; 2 Intervention and Cell Therapy Center, Peking University Shenzhen Hospital, Shenzhen Peking University-The Hong Kong University of Science and Technology Medical Center, Shenzhen, China; 3 Institute of Martial Arts and Traditional Ethnic Sports, Beijing Sport University, Beijing, China; 4 Department of Infectious Disease, Center for Liver Disease, Peking University First Hospital, Beijing, China; 5 Realbio Genomics Institute, Shanghai, China; 6 Health Management Center, Peking University Shenzhen Hospital, Shenzhen, China; 7 Department of Gastroenterology, The Second Affiliated Hospital of Guangzhou Medical University, Guangzhou, China; University of Illinois, UNITED STATES

## Abstract

Recent evidence suggests that athletes have microbial features distinct from those of sedentary individuals. However, the characteristics of the gut microbiota in athletes competing at different levels have not been assessed. The aim of this study was to investigate whether the gut microbiome is significantly different between higher-level and lower-level athletes. Faecal microbiota communities were analysed with hypervariable tag sequencing of the V3–V4 region of the 16S rRNA gene among 28 professional martial arts athletes, including 12 higher-level and 16 lower-level athletes. The gut microbial richness and diversity (the Shannon diversity index (*p* = 0.019) and Simpson diversity index (*p* = 0.001)) were significantly higher in the higher-level athletes than in the lower-level athletes. Moreover, the genera *Parabacteroides*, *Phascolarctobacterium*, *Oscillibacter* and *Bilophila* were enriched in the higher-level athletes, whereas *Megasphaera* was abundant in the lower-level athletes. Interestingly, the abundance of the genus *Parabacteroides* was positively correlated with the amount of time participants exercised during an average week. Further analysis of the functional prediction revealed that histidine metabolism and carbohydrate metabolism pathways were markedly over-represented in the gut microbiota of the higher-level athletes. Collectively, this study provides the first insight into the gut microbiota characteristics of professional martial arts athletes. The higher-level athletes had increased diversity and higher metabolic capacity of the gut microbiome for it may positively influence athletic performance.

## Introduction

The human gut microbiota is considered an indispensable “organ” due to its immense impact on human health, including on the physiology, immune function, nutrition and metabolism of the host. Numerous studies have suggested that the gut microbiome may be related to the development of a wide range of disorders, such as obesity, inflammatory bowel diseases, autism and tumours [1–4]. Dynamic changes in the gut microbiota may be associated with factors such as genetics, age, dietary habits and antibiotic usage. Furthermore, recent studies have provided evidence supporting the prediction that exercise is an important factor for modulating gut microbial communities and might be an important new aspect of microbiota research [5].

Exercise-microbiota interaction studies are just emerging and the underlying mechanisms by exercise influence gut microbial communities are quite unknown nowadays[6]. Still, the preliminary studies revealed that exercise’s impact on gut microbiota is key to microbial richness and diversity [7,8]. Choi *et al*.[7] reported that the gut microbiota taxa could be found more frequently in exercised mice than in sedentary mice. Additionally, the taxon order *Lactobacillales* was more abundant, whereas *Erysipelotrichaceae bacterium C11_K211* was decreased in the exercised mice compared with that in the sedentary mice. Queipo-Ortuno *et al*. [8] reported that the gut microbial taxon *Lactobacillales* was more abundant in the exercised rat than in the sedentary ones. In addition to murine studies, human studies, especially those of professional athletes, are increasing and aim to investigate the unique beneficial and diverse gut microbial characteristics in athletes [9,10]. Clarke *et al*. [9] first reported the characteristics of the gut microbiota in professional rugby players by a high-throughput DNA sequencing-based analysis of their faecal samples. The results indicated that athletes had a higher diversity of gut microbiota than did the sedentary individuals, and 22 distinct phyla were positively associated with protein consumption and creatine kinase. Furthermore, a recent study demonstrated that athletes had a relatively greater abundance of the microbial gene related to amino acid and carbohydrate metabolism and their faecal metabolites were associated with enhanced muscle fitness compared with sedentary individuals [10]. All studies above indicated via direct evidence that professional athletes have microbial features distinct from those of sedentary individuals. However, none of these studies referred to the differentiation of the gut microbiota characteristics in athletes competing at different levels. Our study of 28 enrolled martial arts athletes from a professional training team and used 16S rRNA gene sequencing to determine whether their gut microbiota differed based on competition levels.

## Materials and methods

### Recruitment of athletes

A total of 31 professional Wushu routine martial arts athletes (15 females and 16 males, aged 20–24 years) were recruited from the undergraduates of Beijing Sports University, China. Among them, 12 had achieved the qualifications of “National Elite Athletes” and the other 5 and 14 had obtained the qualification of “National First-class Athletes” and “National Second-class Athletes” respectively, which were issued by the General Administration of Sport of China based on their competitive levels (http://www.sport.gov.cn/n16/n300161/n1401202/n1773613/4983026.html). Based on the qualification criteria, the athletes were divided into two groups according to their qualification: a higher-level group (for the National Elite Athletes, shortened as the “H group”) and a lower-level group (for the National First and Second-class athletes, shortened as the “L group”) for the criteria of the First and the Second-class Athletes is closer in comparison.

The exclusion criteria were the presence of digestive system diseases (e.g., diarrhoea, Crohn's disease, ulcerative colitis), metabolic diseases (e.g., obesity, fatty liver disease, diabetes), alcohol abuse and smoking. Those with a known history of antibiotic use and consumption of probiotic drinks in the past 3 months were also excluded. Each enrolled athlete was asked to fill out the Faeces Pre-collection Questionnaire (FPQ) on their frequency of consumption of food products over the past 3 months, their training durations and their average number of hours of exercise per week (naming exercise load) (S1 File). Each of them signed a written consent for the acknowledgement and approval of applying their data and samples for scientific research purposes. Ethical approval was given by the Ethics Committee of Peking University Shenzhen Hospital. The study conformed to the principles of the Helsinki Declaration.

### Sample collection and DNA extraction

The faecal samples were self-collected in a polyethylene specimen collection system (No.02-544-208, Fisherbrand^TM^, USA) with a portion (approx. 220 mg) placed in a 50 ml faecal tube (No.80.734.311, Sarstedt, German) containing 8 ml of RNA later (AM7021, Life Technologies, USA). The samples were immediately placed on dry ice packs, and finally stored at -80°C. The faecal DNA was extracted with the QIAamp DNA Stool Mini-kit (51504, Qiagen, Germany) following the manufacturer’s instructions.

### 16S rRNA gene amplification and sequencing

Partial 16S rRNA gene sequences were amplified from the extracted DNA with universal primers (341F 5’-CCTACGGGRSGCAGCAG-3’and 806R 5’-GGACTACVVGGGTATCTAATC-3’) targeting the V3–V4 region of the 16S rRNA gene. Each primer was labelled with a sample-specific barcode. The 16S rRNA gene amplification products were sequenced with the Illumina HiSeq 2500 instrument (Illumina, San Diego, CA, USA) with 2×250 base pair (bp) paired-end (PE) sequencing at Realbio Technology (Shanghai, China).

### Data processing and statistical analysis

PANDAseq was applied to assemble the overlapping paired-end reads. A sequence with lengths between 220 and 500 nt with a mean sequence quality score of > 20 was retained for quality control while the data were read with N bases < 3. The high-quality 16s rRNA sequence data were processed to form operational taxonomic units (OTUs) at 97% identity using UPARSE (USEARCH V.7.0.1090)[11]. The bacterial taxonomy assignment was performed using the Ribosomal Database Project (RDP) database and classifier (RDP, http://rdp.cme.msu.edu). α diversity and β diversity indexes were calculated based on the rarefied OTUs using the QIIME V.1.9.1 program. The α diversity that represented the species abundance in the samples was estimated with the Shannon and Simpson diversity indexes. The results were compared between the H group and the L group with the Wilcoxon rank-sum test in R3.1.0. β diversity was used as a measure of the microbiota structure between groups. The results of the weighted UniFrac distance matrices were plotted in the principal coordinate analysis (PCoA), analyses of similarities (ANOSIM) and UniFrac Heatmap using R3.1.0. Different abundant bacterial taxa among the groups were identified using the linear discriminant analysis (LDA) effect size (LEfSe, V.1.0) [12]. Only those taxa that obtained a log-LDA score >2 were ultimately considered. FDR q Value was calculated in R using the p. adjust function [13]. Differentially abundant microbial taxa associated with possible confounders (e.g., gender, age, BMI, and dietary habits) based on Spearman’s correlation (*p* <0.05) were evaluated using generalized linear model (GLM).

Phylogenetic Investigation of Communities by Reconstruction of Unobserved States (PICRUSt;V.1.0.0) provided a number of scripts that could be useful for analysing both 16S rRNA gene relative abundances and the predicted metabolic data [14]. The protein sequences of genes in the merged gene catalogue were aligned to the Kyoto Encyclopedia of Genes and Genomes (KEGG) bioinformatics database (8th KEGG release, December 2014).

All quantitative variables were expressed as the means ± standard deviation. If some parameters were not normally distributed, nonparametric analysis was used. Quantitative and qualitative differences between higher-level and lower-level groups were analszed using Student’s t or Mann–Whitney tests for continuous parameters and chi-square or Fisher’s exact tests for categorical parameters, as appropriate. Spearman’s rank tests were used to analyse associations between single genus abundance and the exercise load. Statistical analyses were performed using R3.1.0. All *p*-values reported were two-sided, and *p* <0.05 was considered statistically significant.

## Results

### Characteristics of the H group and the L group

Among the 31 enrolled martial arts athletes, 3 were then excluded due to their antibiotic use, consumption of probiotic drinks or diarrhoea. The remaining 28 were analysed, including 12 athletes in the H group and 16 athletes in the L group. The demographic characteristics of the two groups are summarized in **Table 1**. No distinct differences were detected according to gender, age, BMI, the training duration (years) or overall dietary habits. However, the exercise load (hours/week) of H group was significantly higher than that of the L group (29.25±9.48 vs. 16.63±6.82 hours/week, *p* = 0.002, **Table 1**).

**Table 1:** The characteristics of the enrolled athletes.

Characteristics	Higher-level group (n = 12)	Lower-level group (n = 16)	*p*-value
**Gender distribution, males**	41.67% (5)	50% (8)	0.718
**Age (years)**	20.08±1.83	20.19±1.22	0.475
**BMI (kg/m**^**2**^**)**	22.24±2.12	21.72±1.21	0.61
**Training duration (years)**	11.17±1.75	9.0±3.50	0.066
**Exercise load (hours/week)**	29.25±9.48	16.63±6.82	0.002
**Dietary Habits (n)**			
**Dairy products (Fr/S/O/N)**	0/3/5/4	0/5/7/4	1
**Fried and fatty food (Fr/S/O/N)**	0/0/5/7	0/0/7/9	1
**Refined carbohydrates (Fr/S/O/N)**	2/0/9/1	2/4/6/4	0.14
**High protein foods (Fr/S/O/N)**	5/7/0/0	3/13/0/0	0.231
**Fruits and vegetables (Fr/S/O/N)**	5/6/1/0	7/4/5/0	0.3
**Protein supplementation (Fr/S/O/N)**	6/6/0/0	6/10/0/0	0.702

Quantitative variables are expressed as the means ± standard deviation. Exercise load, the average number of hours of exercise per week; BMI, body mass index; the weekly frequency of consumption of each kind of food was defined as follows: Fr: Frequently (consuming special food more than once per day); S: Sometimes (4–6 times per week); O: Occasionally (1–3 times per week); N: Never (less than once per week).

### The gut microbiota of the H group is more diverse than that of the L group

A total of 1004541 (1.0 million) high-quality 16S rRNA gene reads were obtained from the 28 samples of the two groups, with a mean of 35880 ±1724 reads per athlete. The 28 samples clustered a total of 634 OTUs at a 3% dissimilarity cut-off, with 61–251 OTUs per sample (S1 Table).

**Fig 1:**
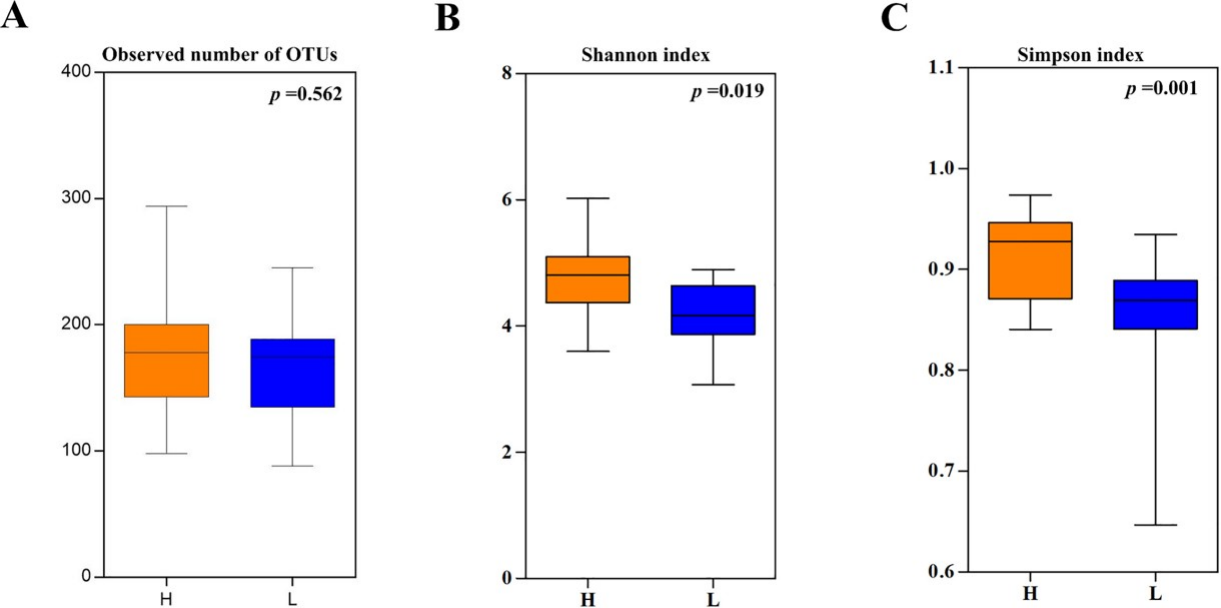
The α-diversity indexes of the faecal microbiota in the samples of the higher-level athletes (H) and lower-level athletes (L). (A) Observed operational taxonomic units (OTUs) (180.8±58.31 vs. 164.4±41.93, *p* = 0.562) between the H group and the L group. (B) Shannon diversity index (4.797±0.722 vs. 4.148±0.543, *p* = 0.019) between the H group and the L group. (C) Simpson diversity index (0.915±0.044 vs. 0.856±0.066, *p* = 0.001) between the H group and the L group.

The OTUs of the H group samples were relatively abundant compared with those of the L group athletes, although there was no statistical significance between the two groups (180.8±58.31 vs. 164.4±41.93, *p* = 0.562). Further analysis of the α diversity of the faecal samples based on the OTU levels revealed that the Shannon (4.797±0.722 vs. 4.148±0.543, *p* = 0.019) and Simpson (0.915±0.044 vs. 0.856±0.066, *p* = 0.001) diversity indexes of the H group were significantly higher than those of the L group (Fig 1). Analysis of these data indicated that the diversity and richness of the gut microbiota in the H group athletes were significantly higher than those in the L group athletes.

**Fig 2:**
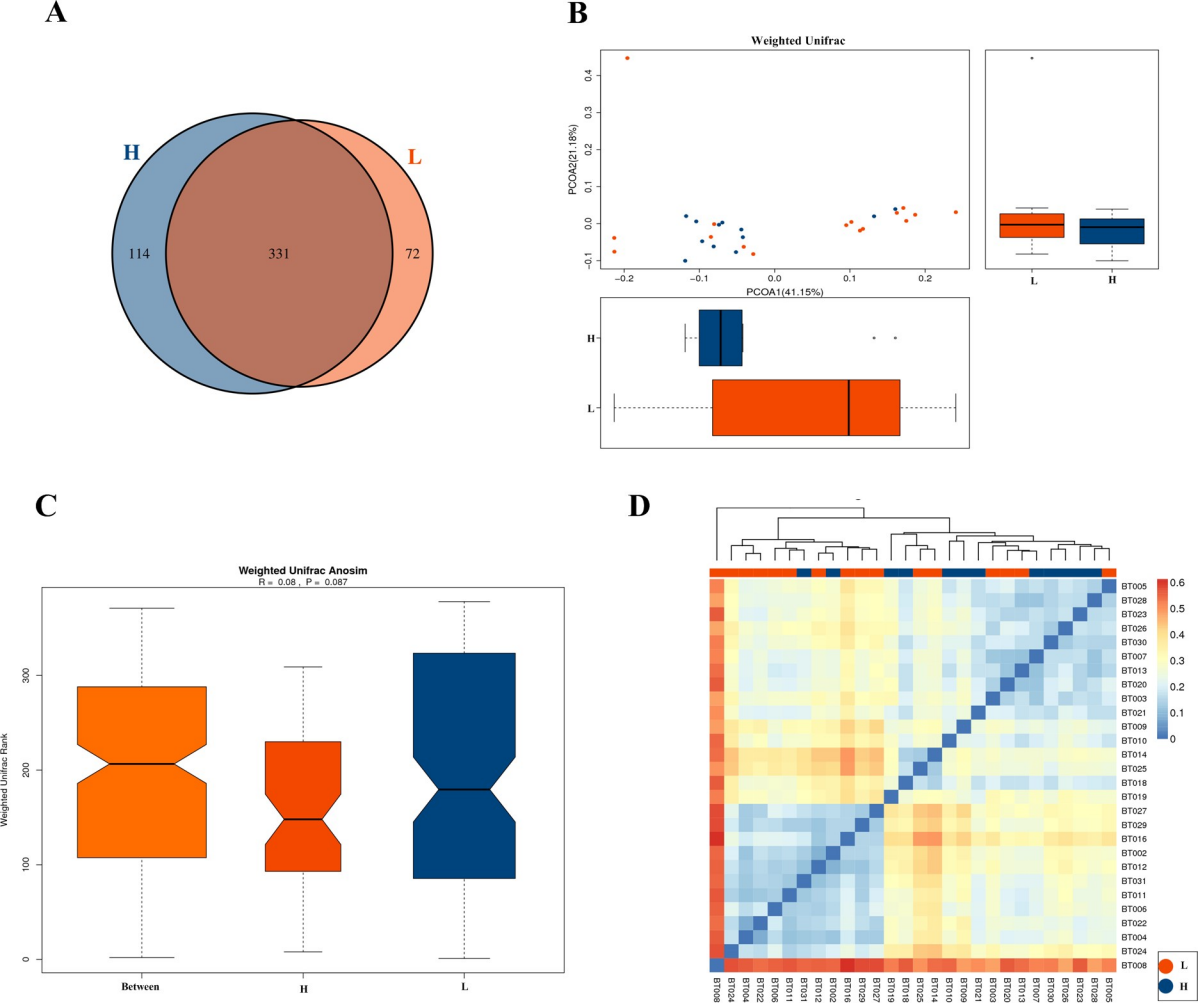
The β-diversity indexes of the faecal microbiota in the samples of the higher-level athletes (H) and lower-level athletes (L). (A) A Venn analysis of bacterial OTUs. (B) PCOA based on the distance matrix of weighted UniFrac dissimilarity of the faecal microbial communities in the H and L groups. The axes represent the two dimensions explaining the greatest proportion of variance in the communities. Each symbol represents a sample: H group (blue) and L group (red). (C) The differences between and within the H and L groups were assessed using one-way ANOSIM. Box plots from the left to the right represent the weighted UniFrac distance between the samples of the H group and the L group, the weighted UniFrac distance of the samples in the H group and the weighted UniFrac distance of the samples in the L group. R values and p values show the community variation between the compared groups. (D) A heatmap with weighted UniFrac phylogenetic distances based on phylotype among samples. The clustering at the top of the figure represents the hierarchical clustering based on the distance between samples. The colour of the grid represents the distance between samples, and the colour corresponds to the icon. The colour from blue to red indicates that the distance between samples increases gradually.

By comparing the OTUs between the two groups, we found that most OTUs were shared by different samples (Fig 2A). To compare the gut microbial structure between the samples of the two groups, the β diversity of their gut microbiota was further assessed. The PCoA plot showed that there appeared a tendency to form 2 clusters, a tight H group cluster (Fig 2B, blue dots) and a disperse L group cluster (Fig 2B, red dots). The results of the ANOSIM analysis indicated that the gut microbiota structure was trend of difference between the in the H group and the L group athletes (R = 0.08, *p* = 0.087) (Fig 2C). Furthermore, the similarity of each sample composition was assessed with a UniFrac heatmap, which suggested a trend of difference between the two groups (Fig 2D).

### Specific microbial taxa differences between the two groups

**Fig 3:**
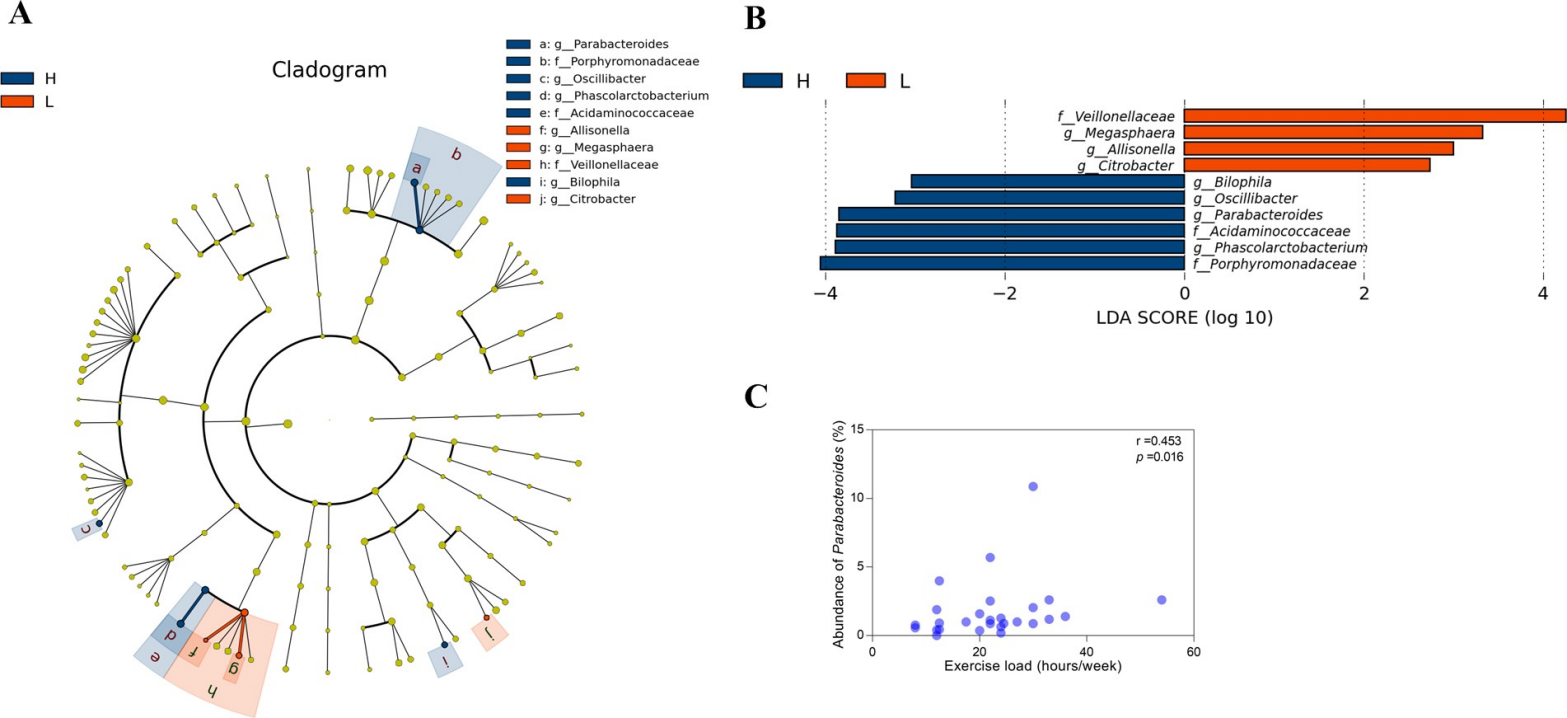
Taxonomic differences in the gut microbiota between the higher-level (H) and lower-level (L) groups. (A) In the cladogram, circles radiating from the inner side to the outer side represent the taxonomic level from family to genus. Blue nodes refer to the dominant bacteria in the higher-level athletes, and the red nodes refer to the bacteria dominant in the lower-level athletes; g, genus; f, family. (B) The histogram of the LDA scores represents the taxa whose abundance showed significant differences between the higher-level and lower-level athletes. The length of each bin, namely, the LDA score, represents the effect size. LDA, linear discriminant analysis; (C) The correlation between the exercise loads per week and the abundance of *Parabacteroides* in the athletes.

A total of 182 microbial taxa were detected in the athletes’ faecal samples, including 2 kingdoms, 11 phyla, 17 classes, 21 orders, 40 families and 91 genera (S2 Table). To further investigate taxonomic differences between the H and L groups, LEfSe analysis was applied. The results suggested that 19 differentially abundant microbial taxa (1 phylum, 1 class, 1 order, 4 families and 12 genera) appeared in both groups (LDA score, Log_10_ >2, S3 Table). Considering that LEfSe analysis does not carry out multiple hypothesis correction when identifying the significantly abundant features in a group, we performed comparative marker selection to identify significantly abundant features in each group using GENE-E (https://software.broadinstitute.org/GENE-E/) [14]. A total of 10 differentially abundant microbial taxa including 3 families and 7 genera were identified between the H and L groups. At the family level, the abundances of *Porphyromonadaceae* and *Acidaminococcaceae* were higher in the H group than in the L group, but that of *Veillonellaceae* was lower. At the genus level, the abundances of *Parabacteroides*, *Phascolarctobacterium*, *Bilophila* and *Oscillibacter* were higher in the H group than in the L group, but those of *Allisonella*, *Citrobacter* and *Megasphaera* were lower (Fig 3A and 3B). Moreover, GLMs were used to model the taxa that were significantly different between the two groups after adjusting for possible confounders. The results indicated that *Acidaminococcaceae*, *Allisonella* and *Citrobacter* were finally excluded for which were significantly correlated with gender, age, BMI, or dietary habits (*p* <0.05; S4 Table). Detailed analyses of the differential microbial abundance of remaining 7 taxa were performed with the Wilcoxon rank-sum test. The statistical results are listed in Table 2.

**Table 2:** Abundant microbial taxa of the faecal samples in the higher-level and the lower-level athletes after adjusting for confounders; only the *p*-values < 0.05 are shown.

Taxon name	Median Abundance (%)		*p*-Value	FDR q Value
	Higher-level group	Lower-level group		
**Family**				
***Porphyromonadaceae***	4.411	1.989	0.008	0.395
***Veillonellaceae***	0.933	4.544	0.003	0.271
**Genus**				
***Parabacteroides***	2.301	0.84	<0.001	0.063
***Phascolarctobacterium***	2.151	0.497	0.028	0.413
***Oscillibacter***	0.661	0.312	0.026	0.413
***Bilophila***	0.25	0.075	0.036	0.413
***Megasphaera***	0.008	0.069	0.04	0.413

Result of the GLMs for abundant microbial taxa based on the group factors (higher-level and lower-level group) and possible confounding factors (gender, age, BMI, and dietary habits) of 28 athletes

FDR, false discovery rate

FDR q Value was calculated in R using the p. adjust function [15].

In this study, because the H group athletes had heavier exercise load, we further evaluated whether the aforementioned abundant microbial taxa could be correlated with the athletic exercise load. The Spearman analysis indicated that only the abundance of the genus *Parabacteroides* was remarkably correlated with the exercise load (Fig 3C; r = 0.453, *p* = 0.016). Furthermore, the abundance of *Parabacteroides* was relatively high for the athletes of the H group reporting 29.25±9.48 hours/week, with a median abundance of 2.301%. The athletes of the L group reported exercising 16.63±6.82 hours/week and a median abundance of only 0.840% *Parabacteroides* (Table 2).

### Functional analysis

**Fig 4:**
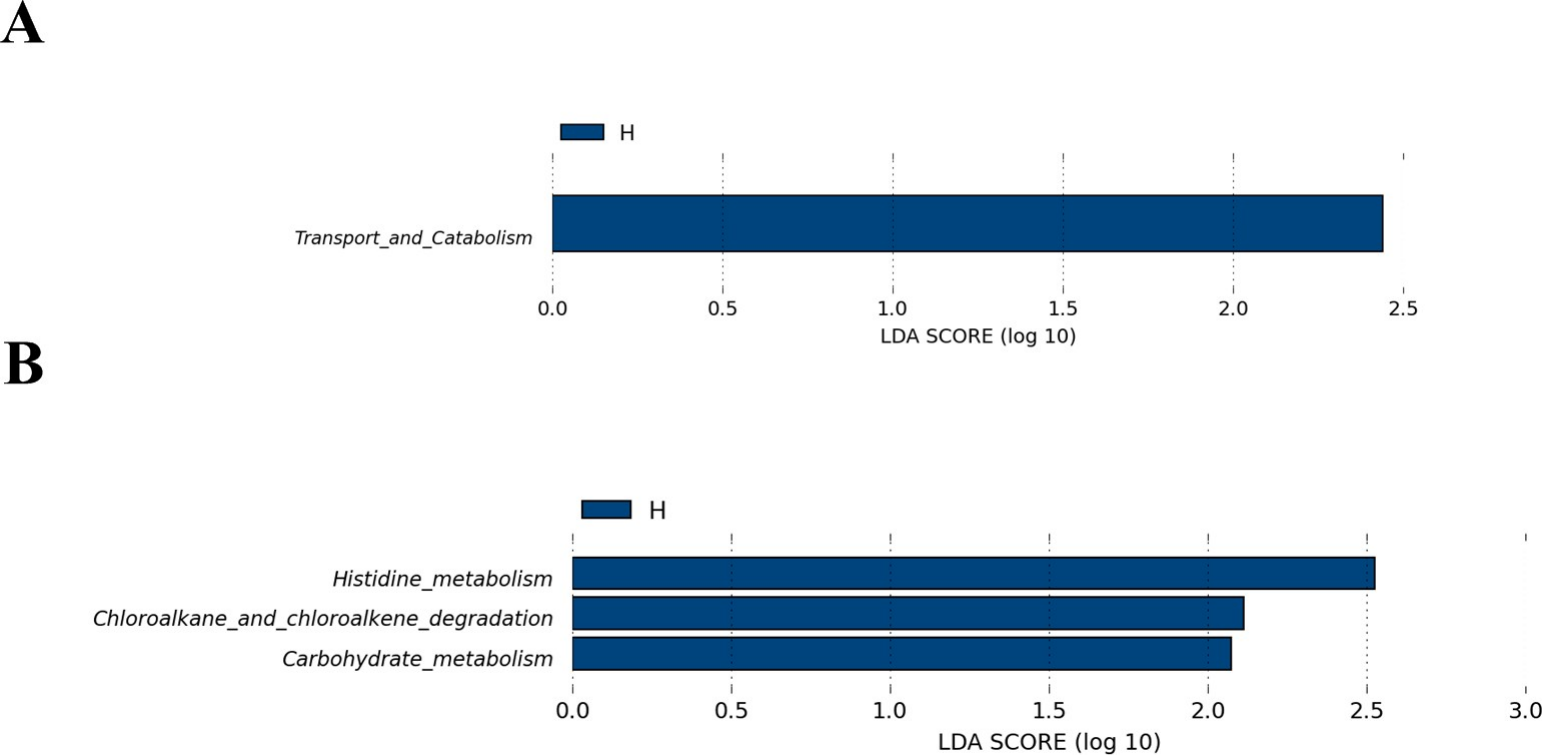
Functional predictions for the faecal microbiome of the higher-level (H) and the lower-level (L) groups. (A) A comparison of the enriched markers on level 2 of the KEGG functional category between the higher-level and lower-level athletes. (B) A comparison of the enriched markers on level 3 of the KEGG functional category between the higher-level and lower-level athletes. LDA, linear discriminant analysis.

The abundances of the functional categories of the KEGG Orthology (KO) were predicted using PICRUSt based on closed reference OTUs. A total of 4 different abundant pathways were identified in the faecal microbiome between the H and L group athletes. In the level 2 KEGG pathway, the microbial gene functions related to transport and catabolism were higher in the gut microbiome of the H group athletes (Fig 4A). In the 3 KEGG pathway, the microbial gene functions associated with histidine metabolism, chloroalkane and chloroalkene degradation and carbohydrate metabolism, were also higher in the gut microbiome of the H group athletes (Fig 4B).

## Discussion

This study provides the first insight into the gut microbiota characteristics of professional martial arts athletes. It was found that the composition of the gut microbiota in the higher-level athletes was significantly different than that in the lower-level athletes. Martial arts is a vigorous sport with several benefits to trainees, such as physical fitness, spiritual health and emotional health and is characterized by vigorous, fast and dynamic movements [16,17]. It requires professional athletes to have a high level of cardiorespiratory fitness and more strength to accomplish series of movements [18,19]. Professional athletes usually have a modified diet with a high consumption of protein and other nutritional components. To largely mitigate the impact of diet on the results, all athletes in this study were enrolled from the same professional martial arts team.

Diversity is important to promote stability and performance in all ecosystems [9]. Microbial diversity may become a novel indicator or biomarker of human health [20]. Loss of biodiversity in the gut microbiota is associated with several disease states such as obesity, metabolic diseases, inflammatory bowel diseases, or recurrent *Clostridium difficile*-associated diarrhoea [1,2,21]. Conversely, increased microbial diversity is linked to increased health in humans, especially in the elderly [22]. Exercise has been reported to be an important factor for increasing gut microbial diversity in humans [9]. Recent studies revealed that the gut microbial diversity in elite athletes was significantly higher than that in sedentary individuals [9,10]. In this study, we found that higher-level athletes had significantly higher gut microbial diversity and richness (α diversity index) than lower-level athletes. Moreover, the higher-level athletes also had a different gut microbial structure (β diversity index) than the lower-level athletes. Prior to this study, Petersen *et a*l. [23] reported the characteristics of the gut microbiota of both professional and amateur cyclists. Their results suggested that the microbial diversity of the majority of professional athletes was higher than that of the amateur athletes, which is consistent with the results of this study. The aforementioned results provided direct evidence that microbial diversity could be positively correlated with professional athletes’ competitive levels.

In our study, a total of 10 differentially abundant microbial taxa including 3 families and 7 genera were identified between the higher-level and lower-level groups. Among the 4 abundant genera in the higher-level athletes, *Parabacteroides* and *Phascolarctobacterium* represented the top two genera. Previous studies have reported that the genus *Parabacteroides*, belonging to the family *Porphyromonadaceae*, was closely associated with exercise and cardiac function, and the species in this family were negatively associated with metabolic disorders [24–26]. The bacterial genus *Phascolarctobacterium* has been reported to produce short chain fatty acids including propionate and acetate, which were associated with the metabolic state, physical exercise and even positive mood of the host [27,28]. The abundant genus *Megasphaera* in the lower-level athletes, belonging to family *Veillonellaceae*, were the most widely studied. It has been reported that *Megasphaera* could be more abundant in chronic inflammatory diseases, such as nonalcoholic steatohepatitis and genital tract inflammation [29], and have the potential to produce high levels of lipopolysaccharides (LPS)[30]. The studies published to date on human individuals showed that elite athletes had beneficial and abundant microbial taxa and that the athletes of different professional types had unique abundant microbial taxa [31]. Petersen *et al*. reported that professional cyclists had a high abundance of the genus *Prevotella*, which was related to amino acid and carbohydrate metabolism pathways. This was significantly correlated with exercise load [23]. Interestingly, in our study, the higher-level athletes had a heavier exercise load than did the lower-level athletes, and we found that the abundance of the genus *Parabacteroides* was positively correlated with exercise load in the martial arts athletes. Thus, it is speculated that the amount of exercise might affect the microbial abundance, and the genus *Parabacteroide* abundance might be correlated with the martial arts athletes’ levels of physical activity [31,32]. The possible mechanism underlying the *Parabacteroide*-exercise interaction influences the athletes' physical activity abilities is still needs.

The professional athletes not only had distinct taxa composition characteristics but also had functional metabolism features in the gut microbiota [9,10]. In our study, further analysis of the functional prediction with the data obtained from the KEGG analysis indicated that the higher-level martial arts athletes had greater metabolic capacity in the gut microbiota than did the lower-level athletes. In agreement with previous studies on professional cyclists or rugby union players [10,23], the microbial gene related to carbohydrate metabolism was remarkably over-represented in the gut microbiome of the higher-level martial arts athletes. Histidine is an important component of muscle carnosine synthesis and its metabolism is closely related to high-intensity exercise performance [33]. Interestingly, our study showed that the pathway related to histidine metabolism was also relatively higher in the gut microbiome of the higher-level martial arts athletes. Indeed, martial arts requires athletes to have more strength to accomplish the series of movements, and the carnosine plays a vital role in maintaining exercise performance by intramuscular buffering capacity. Taken together, our results demonstrate that the exercise performance of martial arts athletes could be influenced by beneficial metabolic functions in the gut microbiota.

There are several limitations in this study. First, an in-depth analysis of the athletes' diets and a cohort of matching non-martial arts athletes was not provided in this study due to various factors. Diet and exercise are the two factors that significantly influence the gut microbiota [9,34]. To further understand the community characteristics of the gut microbiota, future studies are needed and more athlete cohorts and healthy non-athlete cohorts need to be recruited to gather more data on their exercise and diet to analyse the role of diet along with exercise in influencing the gut microbiota taxonomic composition in athletes. Second, metagenomic sequencing analysis, which can provide detailed information about the species of gut microbiota, especially after functional analysis and deeper analysis of the species, was not applied in this study. Such information is necessary for analysing faecal microbial content in higher-level professional athletes. Finally, this is an exploratory study describing the gut community characteristics in professional martial arts athletes. The detailed mechanisms by which exercise-microbiota interactions may influence athletic metabolic abilities and performance warrant further investigation.

## Conclusions

This preliminary study provides the first insight into the gut microbiota characteristics of professional martial arts athletes and we found that higher-level athletes have increased diversity and higher metabolic capacity of the gut microbiome than do lower-level athletes. Further investigations will be important for understanding how abundant microbial taxa such as *Parabacteroides* may respond to exercise and, in turn, positively influence the metabolic abilities and performance of athletes. The aforementioned results also provide opportunities for researchers to generate interesting hypotheses, such as if the gut microbiota characteristics in humans can be applied to predict athletic performance. If so, multiple methods can be used, such as intense training, sports nutrition intake and even probiotics, to modify the gut microbiota to improve athletic performance.

## Supporting information

S1 File Faeces pre-collection questionnaire (FPQ). (PDF)

S1 Table The statistics of OTUs for 28 fecal samples by random selection of 10,671 reads from each sample. (XLSX)

S2 Table Relative abundances of microbial taxa for 28 fecal samples. (XLSX)

S3 Table Taxonomic differences of the gut microbiota between the higher-level (H) and lower-level (L) groups. (XLSX)

S4 Table GLMs for assessing differentially abundant microbial taxa associated with possible confounders based on Spearman’s correlation. (XLSX)
