# Supplementary material for: Characteristics of the gut microbiota in professional martial arts athletes: A comparison between different competition levels
Source: PLoS One. 2019 Dec 27;14(12):e0226240. doi: 10.1371/journal.pone.0226240 (PMC6934331; doi:10.1371/journal.pone.0226240)
Supplement: S1 File — (PDF) [file pone.0226240.s001.pdf]

Faeces Pre-collection Questionnaire

Dear athlete:

Exercise, affecting the state of human health in many ways, and health are one of the main parts of the "Health 2030" strategy. In recent years, it has been considered that sports can affect the diversity of gut microbiota. And different intestinal microorganisms participating in the metabolism of the body are also influential in the exertion of sports skills at the same time. This questionnaire is aimed to analyze the gut microbiota of professional martial arts athletes.

Please read the following questions carefully and answer them truthfully according to your real situation. We promise that this questionnaire will only be used for research and that all information given will be kept strictly confidential. We sincerely thank you for your participation.

Research Group on "Characteristic Analysis of Gut Microbiota of Chinese Martial Arts Athletes"
12 December 2017

Instructions: 1. Please draw "√" in front of your choice, or fill in blanks with appropriate answers.
2. If there is no special explanation, each question has only one answer.

Basic Information

Name: ________ Gender: ________ Age: ________ Height: ________
Weight: ________ Native place: ________ Contact information: ________

Competitive level: □National elite athlete □National first-class athlete □National second-class athlete
Training duration (years):________
Exercise frequency (How many times per week):________
Exercise load (hours):________
Birth mode: □Natural labour □Caesarean (C-) section □Do not know

Status information

1. Consume dairy products (such as pure milk, ect. except yogurt)
   A. Once or more than once per day
   B. 4-6 times per week
   C. 1-3 times per week
   D. Less than one time per week
   E. Never

2. Consume potato chips, fried puffed food

A. Once or more than once per day
B. 4-6 times per week
C. 1-3 times per week
D. Less than one time per week
E. Never

3. Consume lean meat, fish, shrimp, poultry
A. Once or more than once per day
B. 4-6 times per week
C. 1-3 times per week
D. Less than one time per week
E. Never

4. Consume bean products
A. Once or more than once per day
B. 4-6 times per week
C. 1-3 times per week
D. Less than one time per week
E. Never

5. Consume coarse grains such as corns, sweet potatoes, sorghums, etc.
A. Once or more than once per day
B. 4-6 times per week
C. 1-3 times per week
D. Less than one time per week
E. Never

6. Consume a high-sugar diet (candies, chocolates, biscuits, cakes, juice, carbonated drinks, milk tea, etc.)
A. Once or more than once per day
B. 4-6 times per week
C. 1-3 times per week
D. Less than one time per week
E. Never

7. Consume fresh fruits and vegetables
A. Once or more than once per day
B. 4-6 times per week
C. 1-3 times per week
D. Less than one time per week
E. Never

8. Consume protein supplementation (e.g., whey protein)
A. Once or more than once per day

B. 4-6 times per week
C. 1-3 times per week
D. Less than one time per week
E. Never

9. Do you have a drinking habit and drink at least once per month for 6 months or longer? (If yes, please answer question 9 in detail, if not, please skip to question 10)
A. Yes B. NO

10. What is your drinking frequency?
A: Drink 5-7 days per week, drinking age_____, total years_____.
B: Drink 3-4 days per week, drinking age_____, total years_____.
C: Drink 1-2 days per week, drinking age_____, total years_____.
D: Drink less than 1 day per week, drinking age_____, total years_____.

11. Do you have the following diseases diagnosed by regular hospitals? (Multiple choice)
A. Metabolic disease (hypertension, diabetes or hyperlipidemia)
B. Cardiovascular disease (atherosclerosis, atrial fibrillation or congenital heart disease)
C. Liver diseases (viral hepatitis, steatohepatitis, autoimmune hepatitis)
D. Digestive diseases (Crohn's disease, ulcerative colitis)
E. None of the above

12. Have you experienced any of the following feelings in the past three months? (Multiple choice)
A. Burnout, fatigue.
B. Right upper abdominal discomfort.
C. Anorexia, diarrhea.
D. Jaundice, low fever.
E. Constipation.
F. None of the above

13. Have you bought probiotics drinks in the last three months?
A. Yes (if yes, trade name of the probiotics drinks _____).
B. No.

14. Have you taken antibiotics in the last three months?
A. Yes (if yes, trade name of the antibiotics_____).
B. No.

15. Are you taking some kind of drug for a long time?
A. Yes (if yes, trade name of the drug_____).
B. No
